# Advances and challenges in decellularized adipose tissue based composite hydrogels for adipose tissue regeneration: a review over the last fifteen years

**DOI:** 10.7150/thno.120300

**Published:** 2025-08-30

**Authors:** Lu Cui, Tian-Jie Lyu, Bin Fu, Yubo Wang, Qing Qu, Fanglin Wang, Rui Guo, Jun Fan

**Affiliations:** 1 College of Life Sciences, Institute of Life Science and Green Development, Hebei University, Baoding, 071002, China.; 2 Department of Tissue Engineering, School of Intelligent Medicine, China Medical University, No. 77 Puhe Road, Shenyang North New Area, Shenyang, Liaoning Province, 110122, China.; 3 Baoding Key Laboratory of Cancer and Aging, Hebei University, Baoding, 071002, China.; 4 Vascular Neurology, Department of Neurology, Beijing Tiantan Hospital, Capital Medical University, Beijing, China.

**Keywords:** Adipose tissue regeneration, Decellularized adipose tissue, Composite hydrogels, Soft-tissue defects, Translational challenges.

## Abstract

Adipose tissue regeneration has emerged as a transformative strategy for addressing soft-tissue defects resulting from trauma, oncologic resection, and burn injuries, leveraging adipose tissue's dual role as a dynamic endocrine organ that regulates systemic metabolism. Decellularized adipose tissue (DAT) scaffolds hold significant promise in adipose tissue regeneration due to their unique preservation of pro-adipogenic and structurally preserved extracellular matrix (ECM) components. However, their clinical translation faces bottlenecks, including inadequate compressive modulus, unpredictable biodegradation kinetics and limited neovascularization capacity. This review critically synthesizes methodological advancements in DAT processing, systematically evaluating protocol efficacy in DAT preservation versus immunogenic residue elimination while assessing their translational potential as implant materials. Building upon methodological innovations in DAT composite hydrogel engineering since 2013, this overview concurrently elucidates mechanobiological regulation paradigms governing hydrogel functionality and evaluates crosslinking strategies that optimize structural fidelity. Critical challenges and emerging frontiers are also discussed. The current comparative assessment of material performance metrics may offer new insights for further investigation and translational optimization in DAT based composite hydrogels for repair of soft tissue defects.

## 1. Introduction

Currently, plastic surgeons commonly use autologous adipose tissue for surgical grafts in wound repair 1-5, as well as in various cosmetic reconstructive procedures 2, 6. However, despite its widespread application, this approach is limited by several factors, including a shortage of donor tissue, the risk of liquefaction, and the potential for necrosis 7. To address these challenges, decellularized adipose tissue (DAT) presents a promising alternative 8, 9. DAT represents a valuable renewable extracellular matrix (ECM) that serves as a biological scaffold for autologous and allogeneic transplantation, facilitating tissue growth and regeneration. Moreover, DAT can also facilitate the delivery of therapeutic agents, such as drugs 10, growth factors (GFs), and/or stem cells^11^-^15^. The application of DAT has demonstrated significant benefits in the treatment of various diseases, including genetic defects of the breast and chest wall, such as Poland syndrome, systemic adipose atrophy 16, lipodystrophy combined Crohn's disease 17, and facial deformities like Treacher Collins syndrome 18, as well as Barraquer-Simons syndrome 19. Furthermore, DAT's clinical application potential is further enhanced by its intrinsic adipogenic properties 15, 20, along with the growing recognition of adipose tissue as a critical endocrine organ 21. In summary, DAT not only holds significant promise for various medical applications but also presents a more sustainable solution compared to autologous adipose tissue.

Hydrogels are three-dimensional polymeric networks formed via physical or chemical crosslinking methods, exhibiting high water content that mimics biological tissues, and particularly those derived from biological sources possess inherent biodegradability and are suitable for use in minimally invasive techniques 22. Notably, ECM-based hydrogels have gained increasing prominence in recent years 9, as ECM itself comprises a complex architecture of structural macromolecules, including collagens and fibronectin, as well as proteoglycans like glycosaminoglycans (GAGs) 23. Collectively, these components establish a dynamic microenvironment that regulates cellular adhesion, proliferation, and phenotype maintenance through biomechanical and biochemical signaling 23, which is crucial for supporting tissue growth and regeneration. Thus, the use of ECM-based hydrogels is particularly advantageous in tissue engineering and regenerative medicine.

DAT hydrogels have shown potential in promoting the proliferation and differentiation of stem cells, with the expression of adipogenic genes, such as peroxisome proliferator-activated receptor γ (PPARγ), CCAAT/enhancer binding protein α (C/EBPα), and lipoprotein lipase 8. However, DAT hydrogels face several limitations that impede their efficacy in Adipose tissue regeneration. These include inadequate mechanical properties, instability, and insufficient vascularization, which collectively hinder the formation and functionality of engineered adipose tissue 24, 25. The difficulty of large-scale adipose tissue regeneration is exacerbated by hypoxia in the core regions, which induces apoptosis. To address these issues, it is crucial to establish adequate vascularization, as this is essential for supporting long-term cell proliferation and differentiation. Furthermore, the long-standing issue of insufficient vascular supply limits its application in the treatment of severe burns, trauma, tumor resection, and soft tissue defects caused by congenital deformities, as it often leads to the resorption and necrosis of newly formed adipose tissue 26. Therefore, overcoming the challenges related to vascularization is vital for the successful application of DAT hydrogels in adipose tissue regeneration.

DAT-based composite hydrogels, including the composite of scaffold materials, the addition of cells, and the addition of active factors such as growth factors may provide new strategies to overcome these challenges and improve the clinical applicability of adipose tissue regeneration. Further, DAT-based composite hydrogels for adipose tissue regeneration are evolving from structural repair to functional regeneration and may become a disruptive platform for metabolic disease treatment in the future. This review systematically summarizes the preparation methods of DAT and its current challenges, with a focused overview of the advances and challenges in DAT hydrogels and DAT-based composite hydrogels for adipose tissue regeneration. Building on this foundation, we propose future directions to bridge critical technological gaps, aiming to optimize the practical utility of DAT and its composite hydrogels in adipose tissue regeneration while providing theoretical foundations for clinical translation.

## 2. Development timeline of DAT

**Figure 1:**
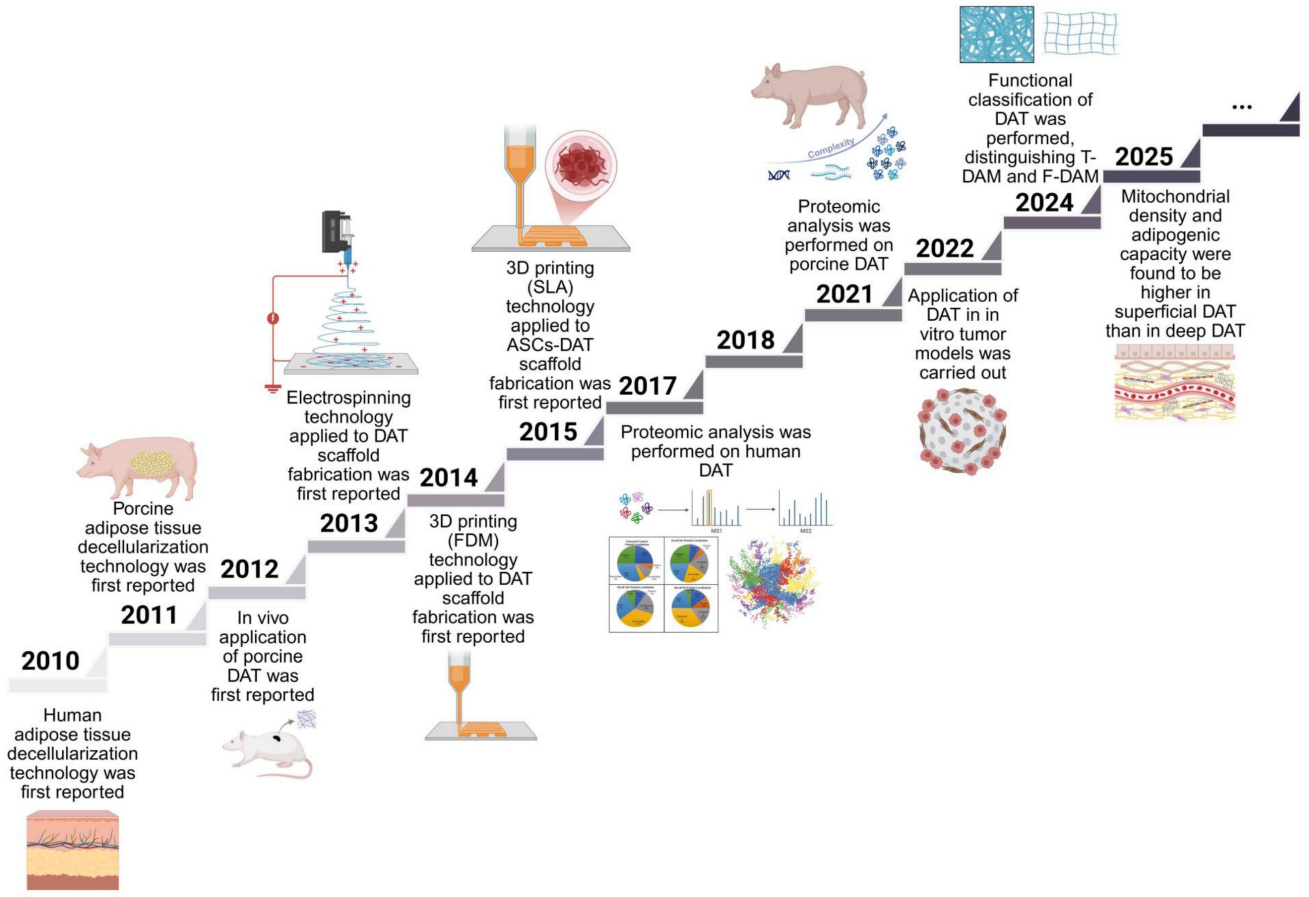
**Timeline of DAT development.** Decellularization technology for human and porcine adipose decellularization technology was first reported in 2010 11, 27 and 2011 28, respectively. Also in 2011, human-derived DAT was first studied *in vivo* 29. In 2012, the first *in vivo* study of porcine-derived DAT was presented 31. In 2013, electrospray technology was first applied to produce DAT microcarriers 37. DAT hydrogels have been used in 3D bioprinting since 2014 42, and the development of 3D printed structures containing DAT bioink with ASCs followed in 2015 43. Recently, an in-depth analysis of the proteomics of DAT derived from human 46, 47 and porcine 44 sources was conducted. Tumor models were 3D printed in 2022 using gelatin methacrylic anhydride compounded with DAT bioinks 60. In 2024, Feng *et al.* introduced a classification scheme for DAT, distinguishing between T-DAM and F-DAM 48. Their study findings suggest that F-DAM was more conductive to promote adipose regeneration. In 2025, Feng *et al.* observed significantly higher mitochondrial density and enhanced adipose regeneration in superficial DAT compared to deep DAT 49. Adapted with permission from 46, copyright 2018 John Wiley and Sons Inc. Created in https://BioRender.com.

The development of DAT scaffolds and advancements in decellularization technologies are presented in the chronological timeline in Figure 1. Pioneering work in 2010 established fundamental protocols, with Flynn 11 and Choi *et al.* 27 independently developing human DAT isolation methods and elucidating its regulatory role in adipose-derived stem cells (ASCs) differentiation. The following year saw methodological diversification, particularly Brown *et al.*'s 28 optimization of porcine DAT processing, which expanded interspecies applicability. This era marked DAT's emergence as a multifunctional, biocompatible scaffold for adipogenesis, vascular engineering, and controlled therapeutic delivery. Notable milestones include Young *et al.*'s 29 2011 demonstration of DAT hydrogel formation through *in vivo* validation, and Lin *et al.*'s 30 groundbreaking application of allogeneic DAT/ASCs composites for rat cavernous nerve regeneration - establishing neural repair as a novel application domain.

In 2012, Choi *et al.* 31 reported decellularized scaffolds of xenobiotic materials, i.e., porcine DAT, for tissue engineering and performed *in vitro* and *in vivo* studies. They demonstrated that the DAT had biochemical and mechanical properties conducive to human cell adhesion and growth, while maintaining biocompatibility, long-term stability, and bioinductivity qualities *in vivo*. Later, Choi *et al.* 32 studied the effects of human DAT on different types of cells and found that the cells exhibited robust propagation, proliferation, and successfully integration into the DAT. These findings indicate that DAT holds great promise as a potential substitute for defective or damaged human tissue. Concurrently, Turner *et al.* 33 engineered DAT microcarriers enabling spatial control of human ASCs differentiation, achieving enhanced neo-adipogenesis and vascularization in rodent models. This period also witnessed innovative crosslinking strategies, particularly Wu *et al.*'s use of hexamethylene diisocyanate and 1-ethyl-3-(3-dimethylaminopropyl) carbodiimide (EDC) to enhance the hydrogel's resistance to enzymatic degradation 34. Importantly, DAT's regenerative versatility was further expanded through synergistic combinations with bone marrow mesenchymal stem cells 35 and hematopoietic progenitor cells 36, establishing multimodal tissue restoration paradigms.

The year 2013 witnessed transformative innovations in DAT scaffold engineering. Pioneering work by Yu *et al.* employed electrospraying technology to fabricate hierarchically porous DAT foams with tunable micro/macropore architectures, demonstrating their efficacy as tunable biomimetic scaffolds for volumetric adipose restoration 37. Importantly, matching the foam stiffness to that of natural tissue enhanced the adipogenesis. Electrospray technology has since been used to fabricate matrix-derived microcarriers using DAT 38-40. Concurrently, Lu *et al.* engineered a heparin-functionalized DAT platform via 1-ethyl-3-(3-dimethylaminopropyl) carbodiimide/N-hydroxy-succinimide (EDC/NHS), enabling sustained basic fibroblast growth factor (bFGF) release to drive vascularized adipogenesis 41. These advancements catalyzed DAT's emergence as a versatile delivery matrix for controlled release of small molecules, growth factors, and immunoregulatory agents, establishing foundational paradigms for precision tissue engineering.

The progressive refinement of decellularization methodologies has positioned DAT as a pivotal biomaterial source for bioink formulation in 3D bioprinting applications. DAT hydrogels have been used in 3D bioprinting since 2014 42. Pioneering work by Pati *et al.* (2015) demonstrated that encapsulating human adipose-derived stem cells (HASCs) within DAT-based bioinks significantly enhanced cell differentiation potential, and soft tissue regeneration 43. Recent advances in proteomic profiling have systematically characterized species-specific DAT compositions (human vs. porcine), providing a molecular blueprint for rational scaffold optimization 44, 45. Emerging evidence emphasizes the method-dependent nature of decellularized matrix proteomes, where collagenase-assisted processing improves detection sensitivity for low-abundance regulatory proteins 46, 47.

Building on these insights, Feng *et al.* (2024) introduced a functional classification of decellularized adipose matrix (DAM), distinguishing between tough decellularized adipose-derived matrix (T-DAM) and fine decellularized adipose-derived matrix (F-DAM) 48. Their work demonstrated that only F-DAM, characterized by its loose fiber architecture and enrichment in matrix / adipogenic proteins, effectively supports adipose tissue regeneration. This structural dichotomy offers a compelling explanation for the previously observed heterogeneity in adipogenic outcomes following DAT implantation. Subsequently, their study (2025) demonstrated that superficial DAT exhibits a higher mitochondrial density and adipogenic capacity compared to deeper layers 49. Critically, they showed that the regenerated fat induced by DAT remained structurally stable and biologically viable for up to one year *in vivo*, with a substantial proportion of adipocytes persisting long-term. These findings provide robust preclinical evidence supporting the long-term regenerative efficacy of DAT-based scaffolds.

DAT has emerged as a versatile and bioactive platform for regenerative medicine. It supports the dynamic expansion and functional maintenance of diverse cell types, including adipose stem cells 50-52, fibroblasts 40, 53, hematopoietic progenitor cells 36, hepatocarcinoma cells 54, endothelial cells 55 and others. Beyond cell culture applications, DAT has revolutionized tissue engineering strategies for the reconstruction of epidural adipose tissue 56, treatment of diabetes 57, and the study of stromal-cell metabolism 58. Recent advances also highlight its potential as a delivery platform for controlled and sustained release of therapeutic drugs and bioactive mediators 10, 59. Moreover, DAT provides a more physiologically relevant and human-derived matrix for the design of preclinical animal models and clinical translational studies. Moreover, DAT provides a more physiologically relevant and human-derived matrix for the design of preclinical animal models and clinical translational studies 60. Notably, in 2022, DAT was successfully integrated with GelMA bioinks for 3D bioprinting of tumor models, marking a novel application of DAT in cancer tissue engineering 60. In parallel, DAT-based scaffolds have been increasingly applied in the regeneration of musculoskeletal tissues, including skeletal muscle 61 and bone 24, where its extracellular matrix components and pro-regenerative signals contribute to enhanced tissue integration and functional recovery. These emerging applications point to DAT's broad translational potential across multiple disease contexts, including wound healing 1 and ischemic tissue repair 36. Ultimately, these cumulative advances delineate DAT as a paradigm-shifting biomaterial, establishing novel pathways for human tissue-derived matrix applications in regenerative medicine.

## 3. Decellularization methods for DAT

**Figure 2:**
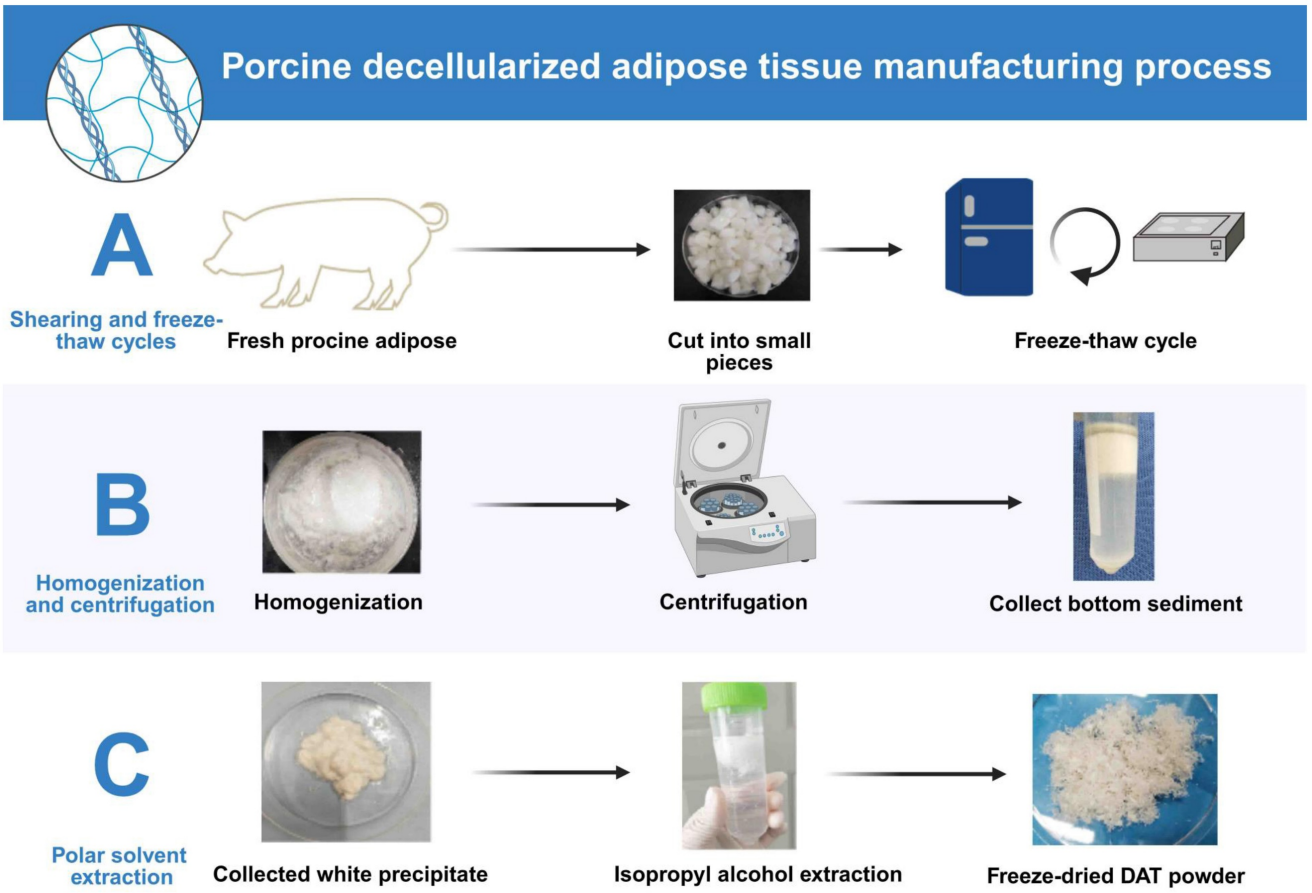
**The decellularization process of porcine adipose. (A)** Fresh porcine adipose tissue is cut into small pieces and subjected to freeze-thaw cycles to lyse cells. **(B)** The tissue is then homogenized and centrifuged to isolate ECM-rich sediment. **(C)** The precipitate undergoes isopropyl alcohol extraction to remove lipids, followed by freeze-drying to obtain DAT powder. This protocol efficiently removes cellular components while preserving ECM integrity. Created in https://BioRender.com.

This section provides an evaluation of various decellularization protocols, some of which have been optimized to maximize the retention of functional ECM components by controlling detergent type and exposure time. Additionally, novel decellularization methods that do not rely on chemical detergents are also discussed. Current decellularization protocols for porcine and human adipose tissue exhibit methodological convergence with species-specific adaptations (Table 1). Based on a comprehensive analysis of the literature and the clinical translational potential of each technique, the most representative and widely adopted decellularization methods for porcine and human adipose tissues were selected. For porcine adipose tissue, the optimized protocol described by Brown *et al.* (Method A) was identified as optimal due to its thorough cell removal, excellent preservation of extracellular matrix (ECM) structure, efficient lipid removal, and strong support for ASCs adhesion and differentiation. For human adipose tissue, the method detailed in Ren *et al.* (Method D) was recognized as the most effective, combining enzymatic and detergent treatments to achieve efficient decellularization while maintaining essential ECM components and biocompatibility. Both protocols stand out for reproducibility, clinical relevance, and frequent application in experimental and translational research. Porcine adipose decellularization is performed at 4 °C or below 37 °C 8, and typically involves sequential steps. First, harvested porcine adipose is cut into fragments or pieces. Then, freeze-thaw cycle decellularization is performed, followed by digestion with an enzyme solution, extraction with polar solvents such as isopropanol, and washing with detergent (Fig. 2). In this process, after each step, the remaining reagent must be washed away with phosphate-buffered saline (PBS) 8, 28, especially when the lipids are extracted using polar solvents. The decellularization process for human adipose is similar to that for porcine adipose. However, in human liposuction is generally performed to obtain the adipose. Moreover, as fresh adipose should be used for decellularization 36, the adipose must be transported to the laboratory on ice, usually within 2 hours 11. During decellularization, some active factors are lost. To minimize this loss, a mild ionic detergent should be chosen. The process is best performed on ice or at 4 °C.

Current standards mandate residual DNA fragments <200 bp with total content <50 ng dsDNA/mg ECM (dry weight) to ensure biocompatibility 62. While enzymatic and chemical decellularization protocols effectively eliminate cellular components without inducing macrophage-mediated pro-inflammatory responses, critical challenges persist regarding ECM component preservation 44. Despite differences in decellular-ization methods, scanning electron microscopy-based and histochemical analyses indicate that the physical structures of the decellularization products obtained using the various decellularization methods are highly similar 44, 46. The urea solvent method developed by Porch *et al.* replicates Matrigel™'s thermal stability and injectability, but proteomic differences between commercial and decellularized matrices suggest that composition variations, related to the decellularization method, may affect clinical translatability 46. Kuljanin *et al.* suggested that collagenase treatment would enhance proteomic coverage of low abundance proteins in DAT 47. Although enzymatic and chemical decellularization methods effectively remove cellular components from the ECM, these processes are often time-consuming and may involve toxic chemicals, which can negatively impact the retention of key proteins and growth factors in the ECM 63. Wang *et al.* 64 recently developed a method for the debulking and decellularization of adipose aspirated tissue using supercritical carbon dioxide, which retained more GFs (such as bFGF and vascular endothelial growth factor (VEGF)) than alternative methods. Qi 65 and Yang 66 *et al.* developed an enzyme-free method for adipose tissue decellularization and demonstrated that DAT promoted adipocyte formation. In a related study, Feng *et al.* found that varying adipogenic effects observed after *in vivo* transplantation may be attributed to differences in the tissue source or preparation methods of DAT 48. Specifically, DAT prepared using conventional enzymatic methods exhibited dense fibrous structures, termed "T-DAM", whereas more delicate fibers were categorized as "F-DAM " 64. Notably, F-DAM derived through ultrasonic separation demonstrated superior capacity for adipose tissue regeneration *in vivo*, indicating that the stromal or F-DAM niche is more conducive to lipid differentiation 48.

## 4. Advantages and limitations of DAT as implant biomaterial

The selection of an appropriate decellularization method is crucial for optimizing the efficacy of DAT in adipose regeneration. Specifically, it affects the preservation of essential bioactive components within DAT, such as VEGF, fibroblast growth factor 2 (FGF-2), and stromal cell-derived factor 1α (SDF-1α). Retaining these factors is vital for enhancing the regenerative potential of DAT in adipose tissue formation, while simultaneously minimizing its inherent limitations. In this section, we examine both the advantages and limitations of DAT in adipose regeneration, including the role of enzymes in the formation of DAT hydrogels and their subsequent impact on adipogenesis. This analysis aims to deepen our understanding of how to fully harness the potential of DAT and enhance its application in adipose regeneration.

### 4.1 Advantages

**Figure 3:**
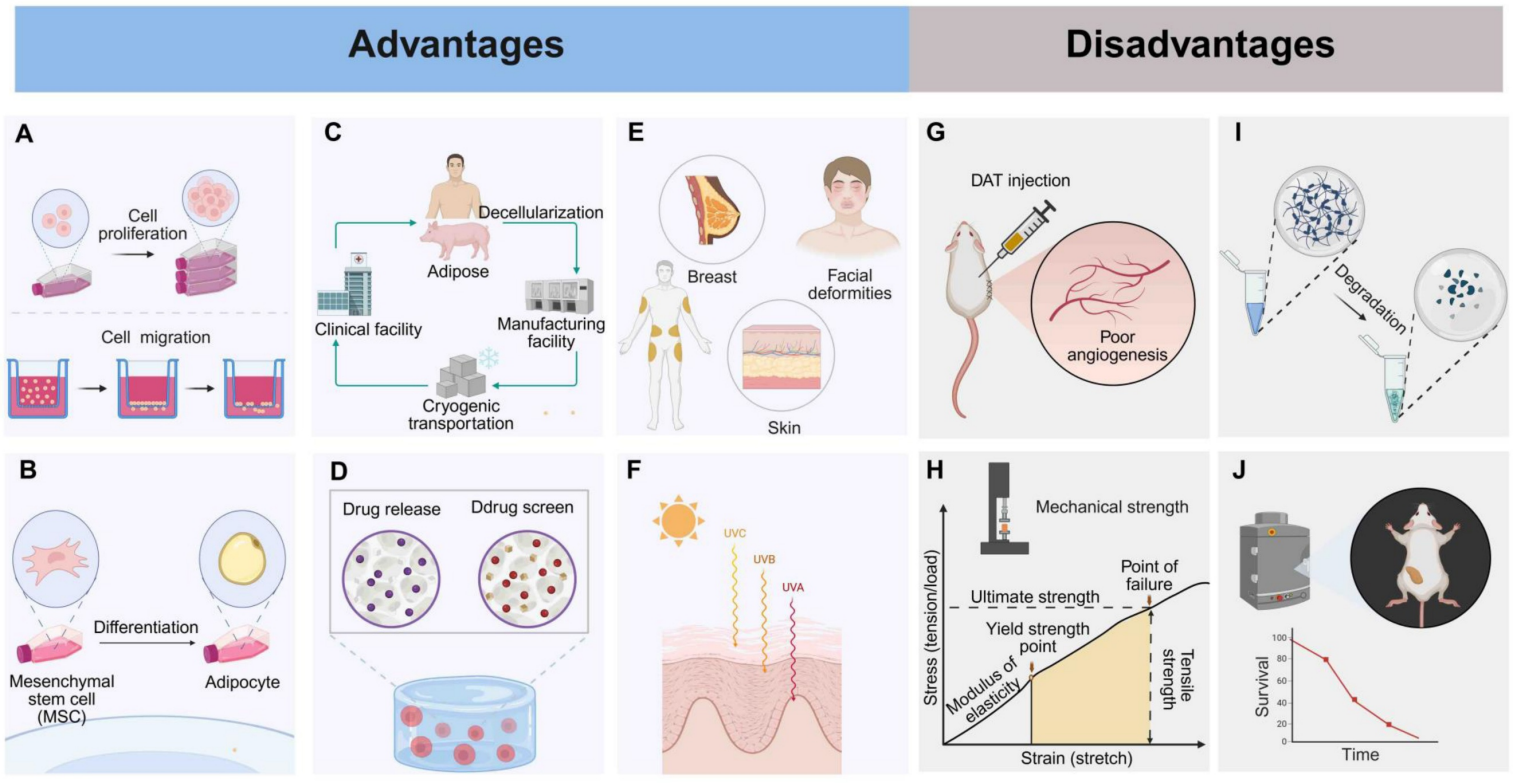
** Advantages and disadvantages of DAT. Advantages: (A)** Promotes stem cell proliferation and migration. **(B)** Promotes adipogenesis. **(C)** Manufacture easily and inexpensively. **(D)** Provides drug delivery and screening scaffolds. **(E)** Fills soft tissue defects. **(F)** Reduces radiation-induced fibrosis. Disadvantages: **(G)** Poor angiogenesis. **(H)** Weak mechanical strength. **(I)** Fast degradation. **(J)** Limited long-term adipogenesis. Created in https://BioRender.com.

An adipose-tissue ECM provides a favorable environment for adipose formation both *in vitro* and *in vivo* 41, 67, 68. After decellularization, DAT-derived scaffold effectively preserves the 3D structure, mechanical properties, and suitable environment of the original adipose tissue. Therefore, this scaffold possesses natural adipogenic properties and can effectively promote the proliferation and migration of adipocytes (Fig. 3A) 8. Proteomic analysis has identified 29 ECM proteins in DAT, which are enriched in adipogenic regulatory functions. Co-culture studies have further demonstrated that DAT can guide mesenchymal stem cell differentiation into functional adipose tissue phenotypes (Fig. 3B) 35. DAT is derived from a wide range of sources (such as medical liposuction waste or porcine adipose tissue), and its decellularization method is relatively simple (Fig. 3C) 8, 69. Currently, a commercialized decellularized human adipose-derived product, Renuva™, is available in the U.S. market, proving that DAT can be produced in a simple and cost-effective manner 70.

Not only does DAT facilitate the delivery of therapeutic agents such as drugs, growth factors, and stem cells (Fig. 3D), but it also shows remarkable advantages in treating various soft tissue defects (Fig. 3E) 41. For example, it has potential in the repair of genetic defects in the breast and chest wall 16, as well as in facial deformities 18, 19. Moreover, protease-digested hydrogels derived from DAT retain essential signaling molecules, such as VEGF, FGF-2, and SDF-1α. These molecules play a key role in orchestrating wound healing by enabling spatiotemporal release that coordinates angiogenesis, fibroblast recruitment, and adipocyte progenitor differentiation 36, 71, 72.

Additionally, DAT scaffolds derived from breast tissue serve as tissue-specific platforms for breast cancer drug screening, demonstrating biomimetic functionality. These scaffolds preserve the native ECM architecture, including high porosity and a nanofibrous structure, which mimic the dynamics of the tumor microenvironment 73. Second, DAT supports radiation damage remediation by enhancing extracellular vesicle-enriched matrix remodeling. This process results in a 65% reduction in fibrosis and restores adipose volume through mechano-transduction-mediated ASC differentiation 74. DAT promotes therapeutic regeneration of radiation-compromised tissues in preclinical models (Fig. 3F) 20. Furthermore, the preservation of VEGF and FGF-2 in supercritical-processed DAT synergizes with adipocyte precursors via PI3K/Akt/mTOR pathway activation, contributing to adipose volume recovery in radiation-damaged models. These multifunctional advancements position DAT as a transformative platform for precision regenerative therapies, effectively bridging the gap between adipose tissue regeneration and personalized oncology applications.

### 4.2 Limitations

DAT as an implant material faces several key challenges. First, DAT scaffolds exhibit limited vascular infiltration, leading to ischemic microenvironments that induce adipocyte apoptosis within 12 hours post-transplantation (Fig. 3G) 75, 76. Surgical procedures such as liposuction worsen this problem by damaging blood vessels and inducing hypoxia-reperfusion injury, necessitating hemodynamic support to promote graft integration and tissue survival 77. Additionally, DAT-derived hydrogels, synthesized through pepsin-mediated decellularization and digestion, exhibit inherent mechanical weaknesses, requiring crosslinking to stabilize their structure (Fig. 3H) 73. Furthermore, unmodified DAT scaffolds degrade rapidly *in vivo*, necessitating controlled degradation strategies to preserve the ECM, prolong bioactivity, and facilitate cellular repopulation and tissue remodeling (Fig. 3I) 78. The proteolytic processing of DAT alters its ultrastructure and composition, which disrupts the bioactivity necessary for effective cell-matrix interactions, leading to insufficient angiogenesis and limited bulk adipose tissue regeneration *in vivo* (Fig. 3J) 79, 80. To address these challenges, DAT-based composite hydrogels have been developed to enhance their performance.

## 5. Cell-free composite hydrogels based on DAT for adipose tissue regeneration

Cell-free composite scaffolds are fabricated by integrating DAT matrix with natural polymers and synthetic biomaterials. Natural materials offer the advantage of superior biocompatibility but are prone to rapid absorption and limited adipogenic induction 67. In contrast, synthetic materials exhibit better mechanical properties and greater stability over time, but they are associated with potential issues such as fibrosis and an increased risk of inflammatory reactions 81.

### 5.1 DAT composite with natural materials

Natural materials complexed with DAT include GAGs, mussel adhesive protein (MAP), silk fibroin (SF), methyl cellulose (MC), and other ECM (Fig. 5A).

#### 5.1.1 DAT complexed with GAGs

**Figure 4:**
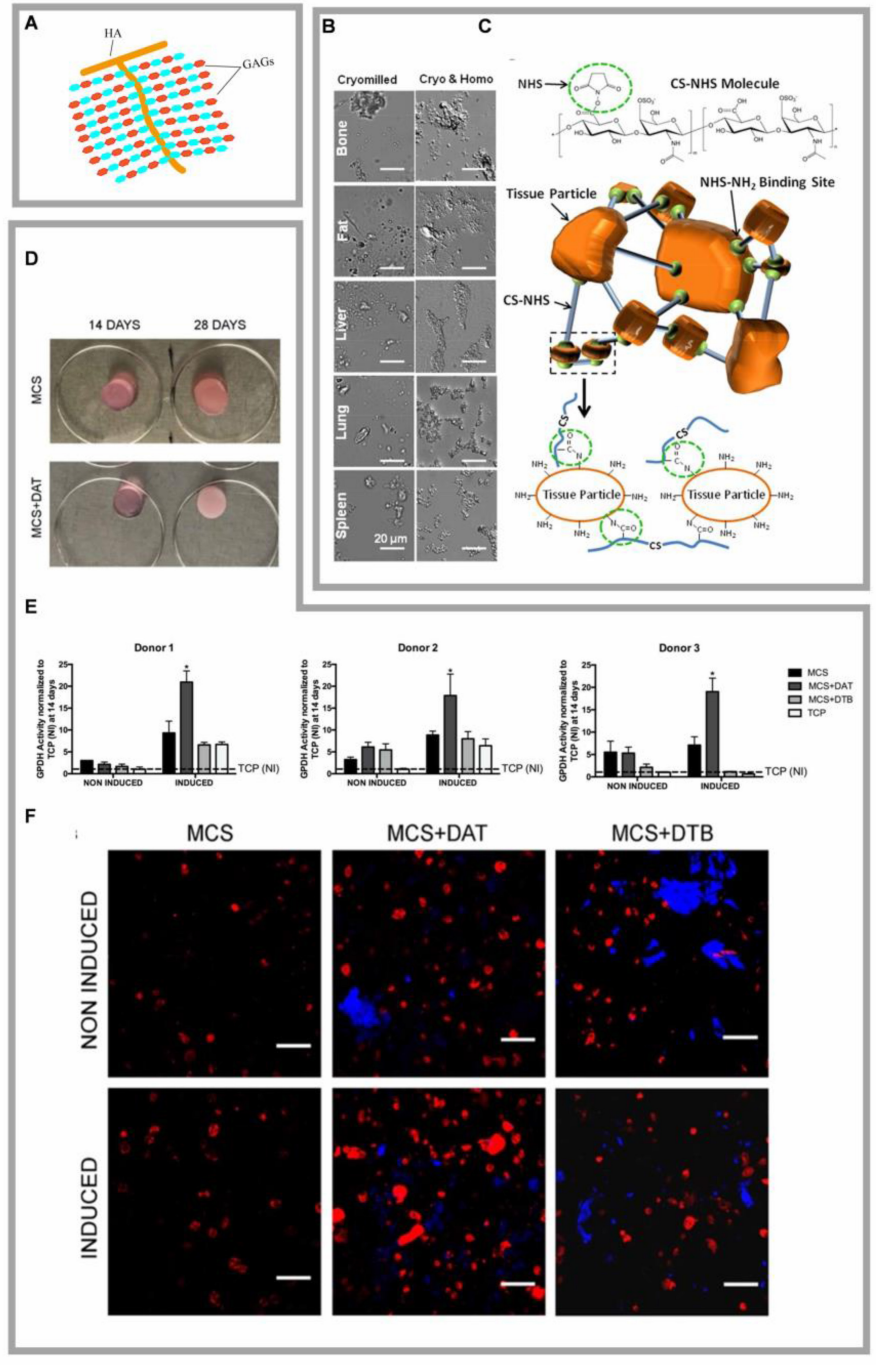
**Highlights of research on DAT compounded with Glycosaminoglycan. (A)** Structure of glycosaminoglycans. **(B)** Differential interference contrast (DIC) images of tissue particles broken down in a cyomill or homogeniser showed that the homogenized tissue appeared smaller (<1 μm) and more uniform. Adapted with permission from 23, copyright 2018 WILEY. **(C)** Chondroitin suladiposee molecules were modified with NHS. Extracellular matrix particle-glycosaminoglycan composite hydrogels for regenerative medicine applications. Adapted with permission from 23, copyright 2018 WILEY. **(D)** Macroscopic images of the MCS and MCS+DAT hydrogels at 14 and 28 days. Adapted with permission from 84, copyright 2019 FRONTIERS. **(E)** GPDH enzyme activity was significantly enhanced in MCS+DAT hydrogels cultured in lipid-forming medium (inducible) for 14 days. Adapted with permission from 84, copyright 2019 FRONTIERS. **(F)** Enhanced intracellular lipid accumulation in MCS+DAT composites induced cells to display a more mature phenotype with larger lipid droplets. Adapted with permission from 84, copyright 2019 FRONTIERS.

GAGs play crucial roles in cellular signaling cascades and tissue homeostasis by modulating the dynamics of bioactive factors through molecular sequestration and controlled release mechanisms (Fig. 4A) 82. The GAG family includes hyaluronan, heparan sulfate/heparin (HS/HP), chondroitin/dermatan sulfate (CS/DS), and keratan sulfate (KS) 83. GAG-ECM composite hydrogels exhibit tunable mechanical properties and programmable ECM particle integration, allowing for tailored performance in both *in vitro* and *in vivo* applications 23. Vince *et al.* engineered an DAT-derived hydrogel scaffold by covalently crosslinking functionalized GAGs with different ECM particulates, circumventing matrix fragmentation and growth factor denaturation caused by enzymatic digestion, while preserving the native DAT's biochemical and ultrastructural features (Fig. 4B, C) 23. Arthi *et al.* demonstrated synergistic adipose formation by combining methacrylated chondroitin sulfate (MCS) with DAT particles, where the MCS-DAT composite microenvironment enhanced adipogenic differentiation of HASCs through tissue-specific matrix signaling (Fig. 4D-F) 14, 84. Cheung *et al.* further validated this approach by encapsulating ASCs in methacrylated glycol chitosan (MGC) or MCS matrices incorporating DAT, achieving enhanced adipogenesis post-implantation 67. Chitosan is a linear polysaccharide derived from the deacetylation of chitin, known for its wound-healing and antimicrobial properties. However, it exhibits limited solubility in water at physiological pH. Glycol chitosan, formed by the reaction of ethylene oxide with chitosan, enhances its solubility in aqueous media across a pH range of 2 to 12. Both MGC and MCS lack inherent biosignals that promote cell adhesion and long-term cell viability. However, when complexed with DAT, the composite hydrogel has been shown to enhance stem cell proliferation and adipogenic differentiation *in vitro*, as well as support adipose tissue regeneration *in vivo* 67. Despite these promising developments, challenges remain in the use of GAGs due to their structural heterogeneity, batch variability, and contamination risks, which limit reproducibility in tissue engineering applications 85. Furthermore, while composite scaffolds incorporating CS have been shown to enhance mechanical properties, mechanical mismatch in soft tissue reconstruction may lead to complications such as scar tissue formation, poor integration, and implant failure 86.

#### 5.1.2 DAT complexed with MAP

MAP, a natural gel secreted by mussels, contains diverse adhesive proteins, making it an ideal biomedical adhesive. MAP is biocompatible, chemically cross-linkable, and integrates long-term with host tissue 87. Additionally, MAP prevents the formation of abnormal collagen protofibrils by binding to collagen molecules. Its lysine/tyrosine residues adsorb cells/factors, enhance platelet adhesion, and induce paracrine effects for healing 88. MAP also inhibits collagenase activity, protecting collagen from degradation 89. Eun *et al.* engineered an adipose regeneration system by integrating DAT with a thermoresponsive bioadhesive hydrogel, synthesized through covalent grafting of bioengineered MAP 90. This MAP modified hydrogels, exhibiting superior water retention capacity, tissue adhesion, and porosity compared to unmodified hydrogels, effectively promoted adipose production by incorporating DAT as a source of adipose-induced and adipose-conducting ECM 90. However, despite its excellent adhesive properties, MAP is prone to oxidation, exhibits low mechanical strength, suffers from poor long-term stability in air, incurs high material costs, and exhibits suboptimal biosafety and biodegradability 91.

#### 5.1.3 DAT complexed with SF

SF is a lightweight (1.3 g/cm³), high-strength (up to 4.8 GPa) protein featuring repetitive hydrophobic blocks and small hydrophilic groups 92, exhibiting biocompatibility and biodegradability 93 for widespread use in tissue engineering applications such as cartilage regeneration 94 and wound healing 95. Alisan *et al.* developed a vascularized adipose tissue regeneration scaffold to promote cell growth and tissue regeneration by incorporating DAT with SF 96. ASCs were differentiated into preadipocytes and preendothelial cells, which were subsequently embedded in a DAT-SF hydrogel at a 1:3 ratio. The preadipocytes showed lipid vesicles formation, while the preendothelial cells were able to form tubular structures within the gel after just 3 days of *in vitro* culture. Angiogenesis was observed after one week of *in vivo* implantation. Hydrogels with a 1:3 (v/v) DAT:SF ratio exhibited superior cell viability 96. However, SF alone demonstrates poor adhesion and proliferation for certain cell types (e.g., neuronal cells) and exhibits low *in vivo* retention efficiency. Therefore, improving the mechanical properties of the DAT-SF composite hydrogels, which are closely linked to cell adhesion, as well as optimizing their degradation rate, remain critical areas for further development (Table 2) 97, 98. In a separate study, Hong *et al.* synthesized SF-GMA hydrogels by introducing glycidyl methacrylate (GMA) to the amine residues of SF and encapsulated cells within these hydrogels 97. The results showed that the mechanical strength of the SF-GMA hydrogels was significantly improved, offering excellent stiffness and durability. Moreover, the degradation time of the hydrogel could be tailored by adjusting the degree and location of methacrylation, offering a customizable approach for hydrogel design 99. These findings offer new insights into the potential of DAT-SF composite hydrogels.

#### 5.1.4 DAT compounded with MC

**Figure 5:**
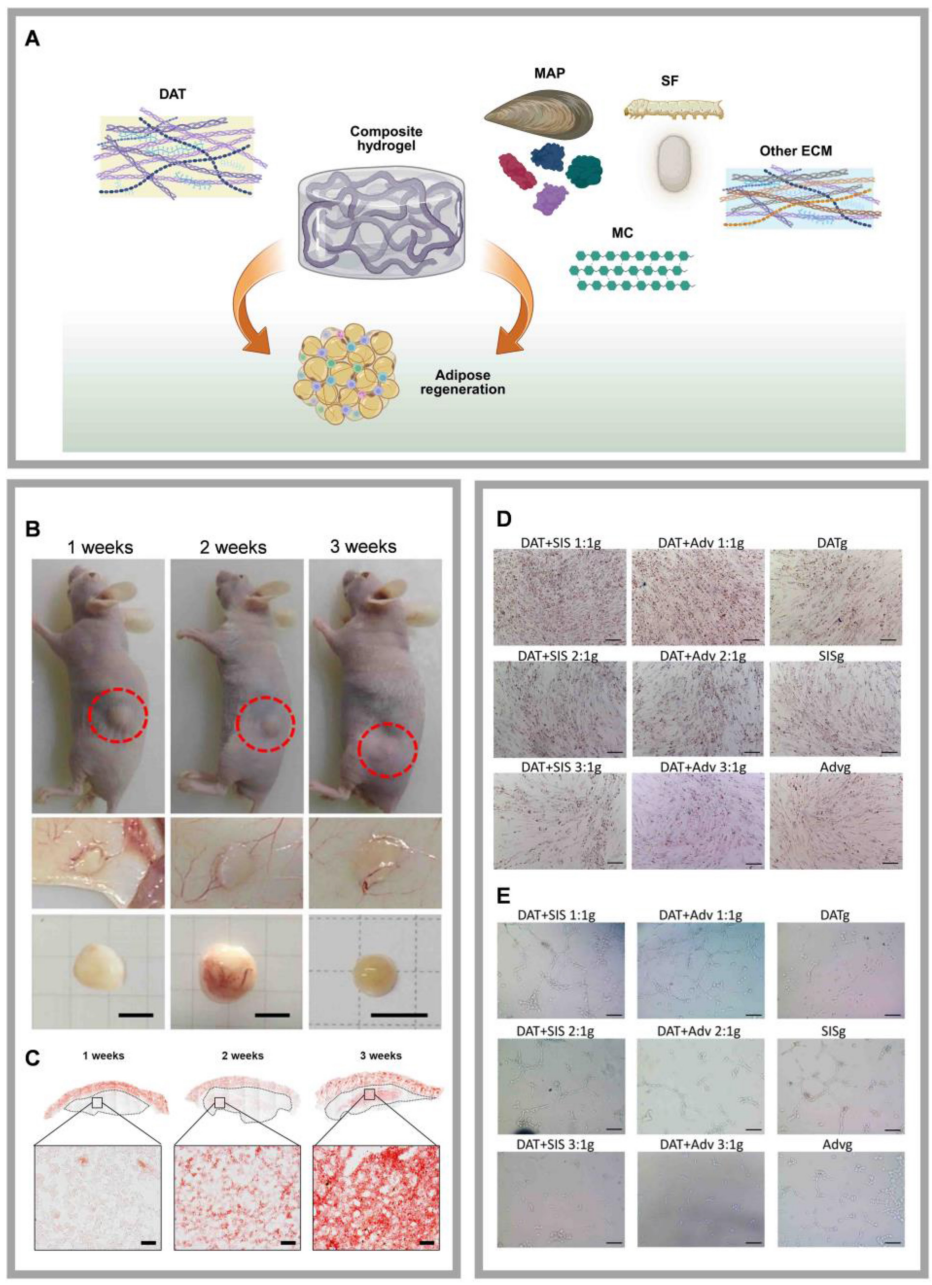
** Highlights of research on DAT composites with natural materials. (A)** Schematic illustration of composite hydrogels formed by combining DAT with various natural materials to promote adipose tissue regeneration. DAT composite with MC enhances adipose regeneration. Created in https://BioRender.com. DAT composite with MC enhances adipose regeneration. **(B)**Typical macroscopic images of *in vivo* grafts observed at 1-, 2-, and 3-weeks post-injection. Adapted with permission from 69, copyright 2017 AMERICAN CHEMICAL SOCIETY. **(C)** *In vivo* grafts at 1, 2, and 3 weeks stained with oil red O. Adapted with permission from 69, copyright 2017 AMERICAN CHEMICAL SOCIETY. DAT composite with ECM achieves adipose regeneration and angiogenesis. **(D)**Typical Oil Red O staining images of lipid formation in ASCs cultured in various DAT-based composite hydrogels. Adapted with permission from 106, copyright 2023 ELSEVIER.** (E)** DAT composite with ECM achieves adipose regeneration and angiogenesis. Typical tube network images of endothelial cells cultured in various hydrogels. Adapted with permission from 106, copyright 2023 ELSEVIER.

Cellulose, an abundant and cost-effective natural polymer, exhibits significant swelling capacity, cytocompatibility, and high viscosity even at low concentrations 100. In contrast, MC, a carbohydrate polymer, is commonly used as a binder or thickener in pharmaceutical formulations due to its ability to effectively maintain the shape of implants 101-103. Jun *et al.* combined DAT with MC to develop a cell-free and scaffold-free adipose tissue regeneration system (Fig. 5B, C) 69. The DAT-MC hydrogel provided an optimal mechanical (3.8 kPa soft hydrogel) and biochemical microenvironment that induced the infiltration and differentiation of host ASCs and adipose tissue macrophages (ATMs), ultimately promoting the formation of new adipose tissue and directly facilitating adipose tissue regeneration. The results suggest that angiogenic factors, such as VEGF and Platelet-Derived Growth Factor BB (PDGF-BB), present in the DAT-MC hydrogel can contribute to angiogenesis by recruiting and activating host endothelial cells. Endothelial cells cultured on soft hydrogels stimulated angiogenesis and network formation more effectively than those cultured on stiff gels 69. Additionally, by modulating the hydrogel stiffness through varying concentrations of MC, the migration of ASCs and post-transplant angiogenesis can be enhanced, effectively promoting adipose regeneration *in vivo* 95. However, while MC offers many advantages, it also demonstrates thermal reversibility, with its viscosity being highly dependent on temperature 104. This characteristic necessitates the inclusion of natural or synthetic modifiers when using MC or its derivatives in 3D printing, to improve the quality and consistency of the final products 105.

#### 5.1.5 DAT composite with other ECM

ECM provides essential structural support and plays a critical role in regulating cellular behavior within tissues. The early development and long-term survival of adipose tissue require the support of an extensive vascular network 106. Decellularized small intestine submucosa (SIS) retains numerous components that promote vascular regeneration, such as connective tissue growth factor (CTGF), bFGF, and transforming growth factor (TGF), all of which facilitate cell proliferation, attachment, and migration, thereby enhancing angiogenesis 107. Similarly, the aortic adventitia (Adv) contains angiogenic and vasoactive factors, including bFGF, which promote endothelial cell proliferation and support the growth of new endothelium 108. We combined DAT with decellularized SIS or Adv and incorporated genipin as a crosslinking agent to develop a decellularized matrix composite hydrogel (Fig. 5D, E) 106. Genipin is a low-toxicity, natural crosslinking agent derived from plants. Crosslinking with genipin enhances the hydrogel's mechanical properties and reduces the inflammatory response 109. The composite hydrogel, fabricated by mixing DAT with SIS or Adv at a 1:1 ratio and crosslinked with genipin, promotes adipose tissue regeneration and angiogenesis by inducing macrophage polarization. Although biocompatible ECMs like SIS and Adv are widely accessible, their inherent degradability limits their functional lifespan, which presents a significant challenge for long-term applications (Table 2). Collagen I, the most abundant ECM protein and a primary product of fibroblasts, provides a structural scaffold that facilitates cell attachment. In addition to promoting the differentiation of ASCs, collagen I also modulates lipogenesis and adiponectin expression 110. However, excessive cross-linking or high concentrations of collagen I may induce a pro-fibrotic response. Notably, Lee *et al.* demonstrate DAT-collagen I-PLCL composites effectively enhance adipogenic differentiation and vascularization 78.

Although natural materials like gelatin, agarose and sodium alginate (SA) have not yet been reported to be compounded with DAT specifically for adipose tissue regeneration, evidence suggests their potential in this and related fields. For instance, Fu *et al.* combined DAT with methacrylated gelatin (GelMA) and methacrylated hyaluronic acid for wound healing 111, Dai *et al.* developed a DAT-GelMA composite bio-ink for colorectal cancer-like organ culture 112, and Grilli *et al.* utilized a GelMA and decellularized plant composite for adipose tissue regeneration 113. These studies highlight the versatility and efficacy of gelatin-based composites in tissue engineering applications. Additionally, the well-established use of agarose as a filler in clinical settings, such as facial aesthetics, further underscores its promising potential for adipose tissue regeneration 114. Comple-mentarily, Rijal *et al.* demonstrated that embedding the antioxidant enzyme catalase within DAT-SA scaffolds can effectively mitigate H_2_O_2_-mediated oxidative stress and hypoxia in large-sized scaffolds 39.

### 5.2 DAT composites with synthetic materials

In addition to compounding with nature materials, DAT has also been compounded with several synthetic materials to enhance adipose tissue regeneration, as discussed following (Table 2).

#### 5.2.1 DAT composite with polyethylene glycol (PEG)

**Figure 6:**
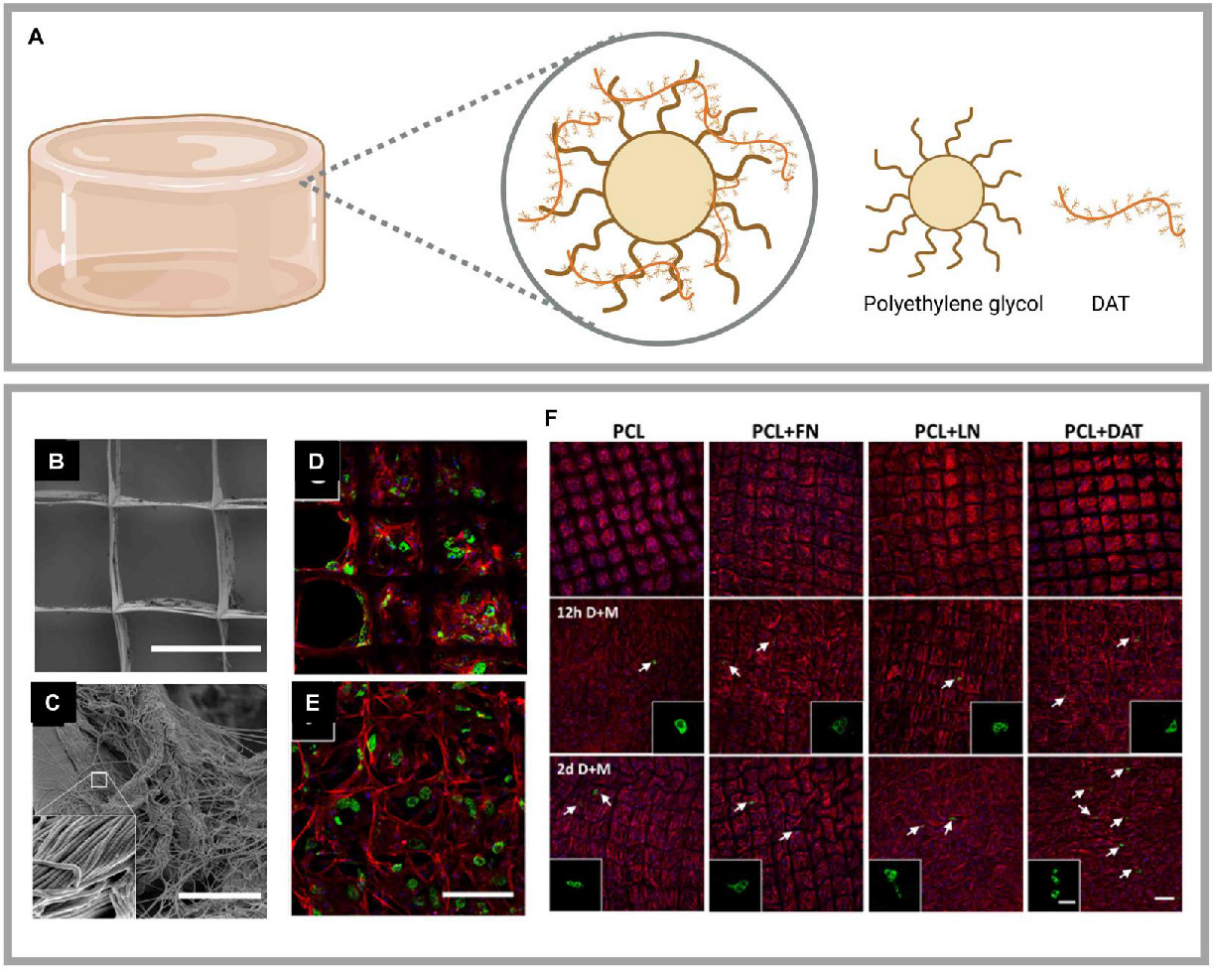
** Highlights of research on DAT composite with synthetic materials. (A)** DAT composite with PEG achieved regenerated soft tissues. Created in https://BioRender.com. DAT composite with PCL achieved regenerated adipose. **(B) (C)** Scanning electron microscopy images of PCL + DAT scaffolds. DAT was uniformly distributed on PCL fibers with a fibrous ultrastructure consistent with that of collagen-rich ECM. Confocal microscopy images of adipocytes after 21 days on **(D)** PCL and **(E)** PCL + DAT scaffolds. **(F)** Confocal microscopy images of the mature adipocytes detected by PLIN1 expression (green), stained actin filaments stained (red), and stained nuclei (DAPI) (blue). Adapted with permission from 120, copyright 2019 AMERICAN CHEMICAL SOCIETY.

PEG is one of the most effective mediators of cell fusion, as it facilitates this process by inducing cell recruitment and modifying the cell membrane 115. The DAT-PEG hydrogel is formed by mixing DAT with synthetic materials through Michael's addition reaction. This hydrogel has been shown to promote the proliferation of HASCs and enhance their lipid differentiation 116. PEG hydrogels offer several advantages, including low protein adsorption, minimal inflammatory response, a well-established safety profile in biological applications, and the ease of incorporating various functional groups. Moreover, the mechanical properties of the DAT-PEG hydrogel, such as its hardness, can be modulated by adjusting the PEG thiol-acrylate content. Liu *et al.* further developed a PEG-modified DAT, positioning it as an ideal biomaterial for allogeneic adipose tissue regeneration (Fig. 6A) 117. This modification significantly improved the M2/M1 macrophage ratio by promoting the secretion of interleukin-10 (IL-10), IL-13, and transforming growth factor β1. Consequently, it reduced immunogenicity and enhanced adipogenesis compared to DAT alone. However, despite the numerous advantages of PEG, it is not without its limitations. At high concentrations, PEG may be toxic 117 and could exhibit immunogenic and sensitizing properties 118. These considerations highlight the need for careful optimization when using PEG-based hydrogels in tissue engineering applications.

#### 5.2.2 DAT composite with poly(ε-caprolactone) (PCL) or poly(L-propylene-co-ε-caprolactone) (PLCL)

PCL is a U.S. Food and Drug Administration (FDA)-approved, biocompatible, and biodegradable synthetic aliphatic polyester, that is widely used in biomedical and pharmaceutical fields 119. Melt electrospinning writing (MEW) is an additive manufacturing technology that can produce continuous, porous fibrous scaffolds through layer-by-layer deposition. Blum *et al.* utilized MEW to fabricate a PCL scaffold and combined it with DAT through non-covalent physical bonding, creating a platform for lipid cell delivery (Fig. 6B-F) 120. However, the relatively slow degradation rate and insufficient mechanical stiffness of PCL *in vivo* limit its application in adipose tissue regeneration.

To overcome these limitations of PCL, researchers have turned to more advanced materials. For example, Lee *et al.* employed 3D printing with a dual-nozzle system to fabricate a hydrogel-PLCL composite scaffold. The hydrogel was composed of DAT and collagen I, while the PLCL component was synthesized via the ring-opening polymerization of L-lactide and ε-caprolactone (CL). This composite scaffold effectively promote adipogenic differentiation and vascularization 78. A key advantage of PLCL is that its mechanical properties—such as flexibility, stretchability, and elasticity—as well as its degradation rate, can be precisely customized by adjusting the ratio of the two monomers. These customizable properties enable PLCL to outperform PCL in soft tissue engineering applications. Therefore, PLCL is considered an ideal alternative to address the mechanical limitations of PCL, with the potential to significantly improve the long-term stability and cell survival of DAT scaffolds 121. However, both PCL and PLCL face common challenges, including central hypoxia and inadequate scaffold porosity that my lead to tissue necrosis. Moreover, the degradation process of both materials is complex, carrying risks such as premature loss of mechanical integrity and the release of toxic byproducts, which wuld potentially compromise the long-term biocompatibility of the implant 119. These issues highlight the need for optimization to improve the efficacy and safety of PCL/PLCL-based scaffolds.

## 6. Cell-based or bioactive factor-infused composite hydrogels based on DAT for adipose tissue regeneration

Establishing functional vasculature is essential for both adipose tissue regeneration and the long-term survival of engineered scaffolds 122. To address this critical challenge, DAT-based composite hydrogels have emerged as versatile platforms for integrating cells or bioactive factors to drive vascular network formation. These systems leverage DAT's inherent biocompatibility and pro-regenerative ECM components while addressing its limitations through strategic modifications. The advantages and challenges of DAT-cell/bioactive factor composites were summarized as following (Table 2).

### 6.1 DAT complexes with cells

**Figure 7:**
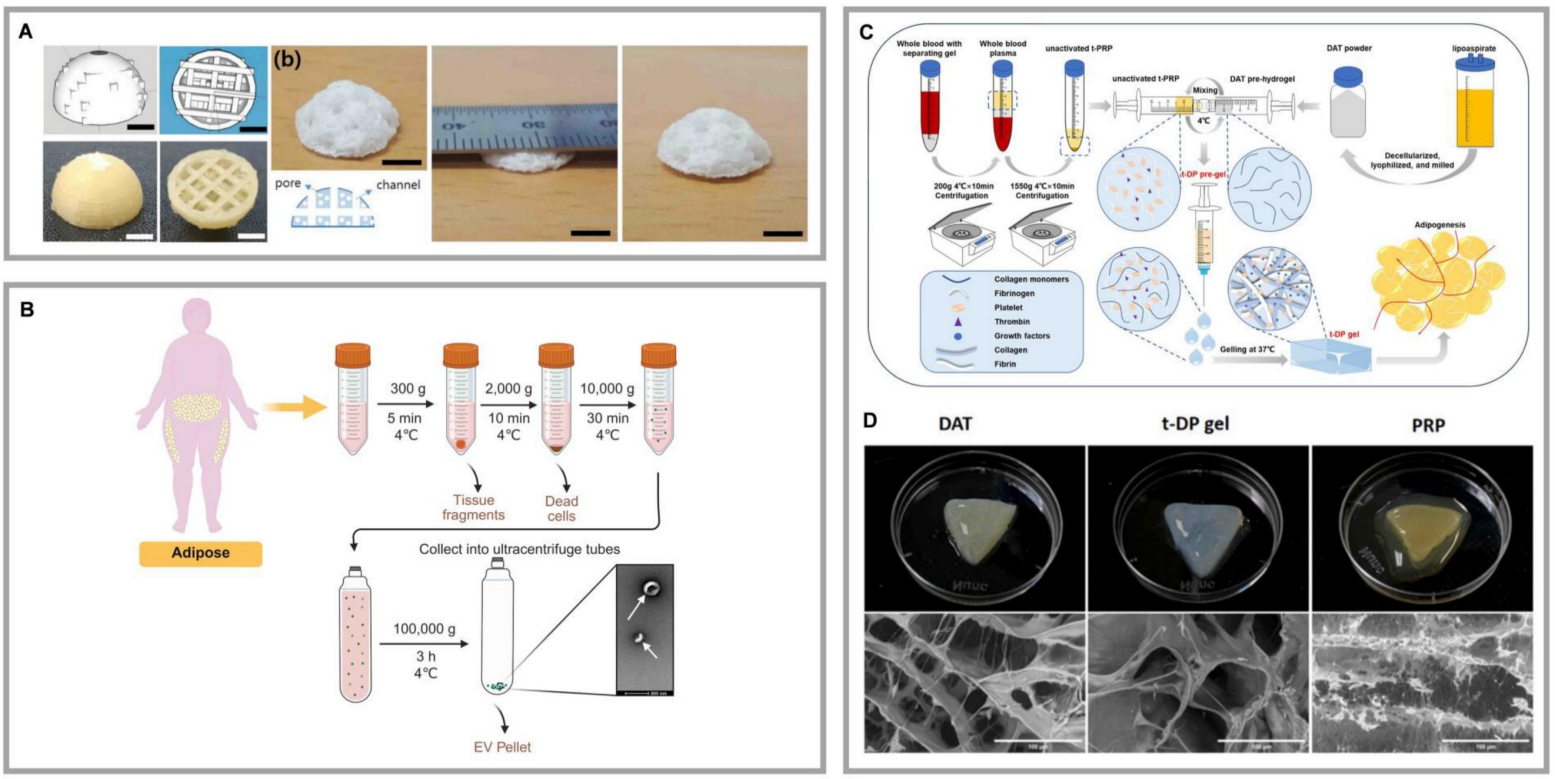
**Highlights of research on DAT composite cell or bioactive factor. (A)** DAT composite PLCL scaffolds enabled regenerated soft tissues. 3D-printed PVA molds and PLCL scaffolds with a porous structure. Adapted with permission from 124, copyright 2021 MDPI. **(B)** Process for the isolation of adipose tissue-derived extracellular vesicles from adipose liquid extracts. Created in https://BioRender.com. **(C)** The process of preparing PRP from whole blood. Adapted with permission from 129, copyright 2025 WILEY-BLACKWELL PUBLISHING LTD. **(D)** Upper panel: photographs of PRP, DAT, and t-DP (t-PRP and DAT gels were mixed at low temperature to obtain t-DP) hydrogels at 37°C. Lower panel: the typical microstructural images of PRP, DAT, and t-DP hydrogels observed under scanning electron microscopy. Adapted with permission from 129, copyright 2025 WILEY-BLACKWELL PUBLISHING LTD.

HASCs exhibit significant adipogenic potential, while human umbilical vein endothelial cells (HUVECs) demonstrate high efficacy in vascular regeneration 123. HUVECs are the primary cell type of vascular endothelial cells and are capable of forming vascular networks. Through interactions between cells and between cells and the ECM, HUVECs promote the formation and maturation of capillaries. Additionally, HUVECs secrete various growth factors and cytokines, such as VEGF and bFGF, which facilitate angiogenesis and endothelial cell proliferation. With excellent adhesion and migration capabilities, HUVECs respond to chemical signals, such as VEGF, guiding their migration to the site of injury and contributing to the formation of new vascular networks 123. The proliferation and adipogenic differentiation potential of preadipocyte cells also provide possibilities for adipose tissue regeneration 123. By forming composites with DAT, the adipogenic properties of ASCs and the angiogenic capacity of HUVECs are further enhanced. Forming composite hydrogels with DAT allows for the simulation of the natural tissue microenvironment, enhancing cell-to-cell synergy, improving oxygen and nutrient supply. This better supply supports long-term cell survival and function, significantly boosting both adipose tissue regeneration and angiogenesis. Zhao *et al.* developed a 3D printing technology that combines human subcutaneous preadipocyte cells with DAT, forming a novel scaffold structure. Additionally, they incorporated this scaffold with aortic decellularized matrix (dECM) and HUVECs, resulting in a composite scaffold that effectively enhances adipose regeneration and angiogenesis 123. Kim *et al.* further explored a composite scaffold made from PCL scaffold, adipose dECM/heart dECM (80/20), and HASCs. This structure demonstrated superior angiogenesis, apoptosis resistance, and adipose tissue formation (Fig. 7A) 124. Zhu *et al.* engineered vascularized adipose tissue by combining silica-expanded capsules with DAT, HASCs, HUVEC cell sheets, and exogenous chemokine CCL2, leading to improved adipogenesis and angiogenesis 125. This integrated approach not only leverages the unique strengths of different cell types and biomaterials but also underscores their potential in advancing complex tissue engineering strategies.

Despite these studies demonstrating the efficacy of DAT-cell composites, challenges remain. Cells within scaffolds may release excessive hydrogen peroxide, leading to oxidative stress, hindering tissue regeneration, and causing tissue damage. To address this, Rigal *et al.* proposed an innovative strategy by encapsulating the antioxidant catalase (AC) in a gel scaffold composed of DAT and high-viscosity SA 39. The DAT-SA-AC hydrogel effectively reduces oxidative damage, promotes tissue growth, and enhances angiogenesis 39. However, despite the compelling evidence for tissue regeneration provided by these studies, the survival and integration of implanted cells remain insufficient, leading to inadequate angiogenesis. Zhao *et al.* improved the survival rate of ASCs using an oxygen-glucose deprivation (OGD) model and significantly upregulated the levels of VEGF and SDF-1α, improving their application in ischemic diseases 122. Additionally, Souga *et al.* discovered that pre-treating ASCs in endothelial growth medium enhanced their pro-angiogenic properties, improving their therapeutic effects on ischemic diseases 126. Nevertheless, excessive hydrogen peroxide release from cells encapsulated in DAT may lead to tissue damage, and the challenges of cell immunogenicity, survival, and integration require further resolution 39.

### 6.2 DAT composite with bioactive factors

The close relationship between angiogenesis and adipogenesis suggests that the limited adipogenic potential of DAT may be due to insufficient neovascularization, which affects ASC's differen-tiation and adipose tissue regeneration. To address this bottleneck, incorporating angiogenic factors into DAT scaffolds has become a key strategy. Liu *et al.* encapsulated VEGF in heparinized DAT (Hep-DAT) hydrogels, significantly accelerating wound healing and angiogenesis by controlling VEGF release. Similarly, Lu *et al.* used EDC/NHS chemical crosslinking to immobilize bFGF on Hep-DAT, effectively promoting angiogenesis and adipose regeneration 41. Human adipose tissue-derived extracellular vesicles (hAT-EVs) have dual regulatory functions: they induce ASC adipogenesis and activate HUVEC proliferation, migration, and angiogenesis 59. Figure 7B demonstrates the isolation process of hAT-EVs. Nie *et al.* integrated hAT-EVs into DAT scaffolds for soft tissue repair, enhancing both adipogenesis and angiogenesis 59, 127.

Platelet-rich plasma (PRP) has shown promise in enhancing angiogenesis through the release of growth factors, but its application is limited by the suboptimal localization of these factors. Zhou *et al.* demonstrated that immobilizing PRP on gelatin microspheres improved the survival of implanted adipose tissue 128. In further developments, Hou *et al.* engineered a thermoresponsive hydrogel system that integrated DAT with temperature-regulated PRP (t-PRP). This composite scaffold exhibited enhanced structural stability under physiological shear stress and sustained release of angiogenic factors (Fig. 7C, D) 129. These studies collectively highlight the synergistic potential of integrating DAT with PRP to enhance adipogenic differentiation and vascularization. However, PRP presents limitations, including variability between donors, which can lead to inconsistent clinical outcomes. Additionally, the presence of leukocytes, platelets, and other particles in PRP may provoke unwanted immune responses. To address these issues, platelet-free plasma (PFP), which contains fibrinogen but fewer platelets, has emerged as a promising alternative. Aurora *et al.* developed a composite scaffold combining a polyethylene-glycolylated PFP hydrogel with a muscle-derived ECM stent, which upregulated angiopoietin-1 and Tie-2 expression, promoting vascular induction 130. Although the combination of PFP with DAT in adipose tissue engineering has not been extensively studied, recent work by Amo *et al.* introduced a novel 3D-printed wound dressing made from a composite bioink consisting of DAT, human dermal fibroblasts, and low-platelet plasma or PRP 131. This bioprinted dressing facilitated the synthesis and release of signaling proteins involved in wound healing and regeneration, creating an optimal microenvironment for tissue repair. Although these bioactivation strategies can be tailored for specific applications, significant barriers to clinical translation remain: high costs, risks of prolonged factor retention, and limitations in controlled release technologies.

## 7. Mechanical properties and structural stability of DAT-based composite hydrogels

DAT hydrogel is a temperature-sensitive gel. Lyophilized DAT exhibits a porous sponge-like structure with a mesh size ranging from 20 to 50 μm and an average porosity of approximately 90% 132. Yu *et al.* reported that at -20°C, the Young's modulus for 50 mg/mL DAT was 3.67 ± 0.38 kPa, and for 100 mg/mL foam, it was 4.00 ± 0.38 kPa. At -80°C, the Young's modulus for 50 mg/mL DAT was 2.42 ± 0.65 kPa, and for 100 mg/mL DAT, it was 4.01 ± 0.46 kPa 37. Wenderott *et al.* reported that the Young's modulus of normal human visceral adipose tissue was 4.48 ± 4.81 kPa, while that of diabetic visceral adipose tissue was 11.50 ± 16.79 kPa 133. In comparison, Kayabolen *et al.* provided data for subcutaneous adipose tissue, reporting an initial modulus (10% strain) of 6.6 ± 1.8 kPa, a transition modulus (20% strain) of 24.5 ± 3.6 kPa, and a final modulus (30% strain) of 87.1 ± 8.7 kPa 96. Furthermore, Lee *et al.* reported the compressive modulus of scaffolds, which were approximately 3.6 MPa for PCL, 122 kPa for PLCL, and 40 kPa for adipose tissue 78. Notably, the Young's modulus of adipose tissue can vary significantly depending on the anatomical location and measurement methods, such as using a universal testing machine or atomic force microscopy 133. This varations highlight that a comprehensive understanding of the tissue's mechanical properties requires consideration of both the specific adipose tissue type and the evaluation technique used. The interactions between cells and hydrogels are influenced by the hydrogel's mesh size and mechanical properties, which are determined by factors such as stiffness, stress relaxation, and the degradability of the surrounding polymer. Specifically, the mesh size affects cell spreading, growth, migration, and the development of the ECM. The mechanical properties of the hydrogel are particularly crucial when cells are encapsulated within it, as they require direct mechanical support to induce phenotypic changes, including migration and other cellular behaviors 134. Consequently, both the mesh size and the mechanical properties of DAT and DAT composite hydrogels are pivotal factors to consider for successful tissue engineering. For example, Table 3 summarizes the Young's modulus and respective advantages of hydrogels composed of DAT and natural materials.

Modifications to the structural stability of a bio-scaffold need to meet stringent criteria, including biocompatibility, porosity, biodegradability, and mechanical suitability 135. Cell infiltration is crucial for enhancing structural stability 90, and hydrogels with high porosity are essential for promoting this, while their mechanical suitability is critical for cell function, as poor stability can lead to cell detachment and death 136. Fibrillar ECM proteins like fibronectin, collagen, and elastin form a fibrous network that displays mechanical asymmetry—stiffening under shear or tensile strain, and softening under compression. This property protects tissues from excessive deformation while regulating cell contraction, migration, proliferation, and differentiation 137. Therefore, maintaining optimal mechanical conditions is crucial for effective use of DAT and DAT-based composite hydrogels in adipose tissue regeneration.

**Figure 8:**
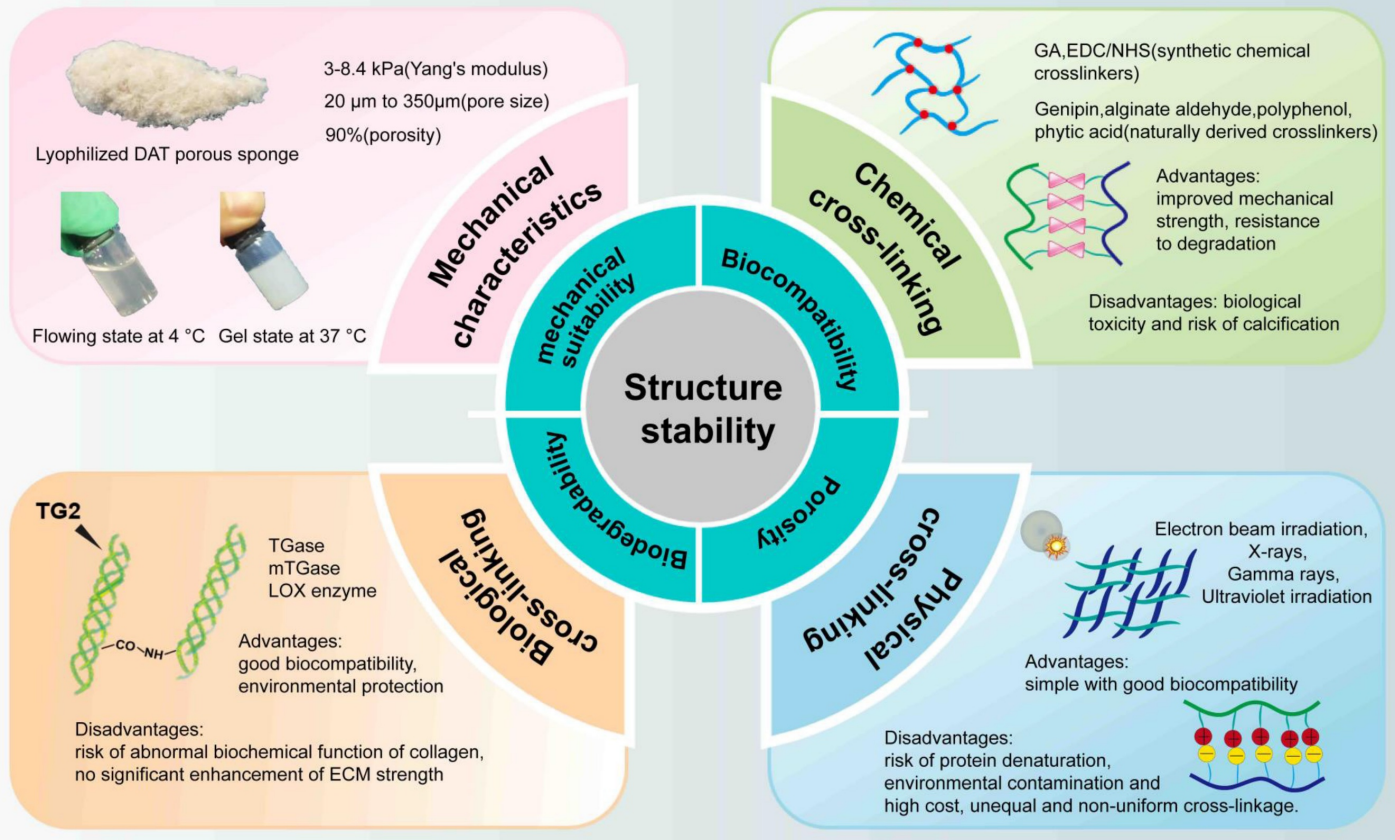
**Stability of the DAT composite hydrogel**.

To enhance the mechanical stability of composite hydrogels, strategies such as crosslinking are commonly employed, including chemical, physical, and biological crosslinking (Fig. 8). Crosslinking can occur through intermolecular or intramolecular bonds, depending on the reaction conditions. This process improves mechanical properties, such as degradation resistance, elasticity and other properties of the polymer characteristics 138. For example, intra-fiber crosslinking can control the stiffness of fibers, resulting in changes to the hydrogel's stiffness without altering its overall structure 137. The following sections disscuss various crosslinking methods for enhancing the mechanical properties of DAT-based composite hydrogels.

Synthetic chemical crosslinkers, such as glutaraldehyde 139 and and 1-ethyl-3-(3-dimethy-laminopropyl) carbodiimide/N-hydroxysuccinimide (EDC/NHS) 140, 141, are commonly used but are associated with certain limitations, including cytotoxicity and the risk of calcification. Additionally, unreacted crosslinkers must be removed prior to implantation, which complicates their practical use 142. In contrast, naturally derived crosslinkers—such as alginate aldehyde from brown algae 143, 144, genipin from gardenia fruit 106, 145, polyphenols from natural sources 146, 147, dialdehyde CMC 148, and phytic acid (PA) from plant seeds 149—are generally more biocompatible and less toxic than their synthetic counterparts 150. However, these natural crosslinkers often suffer from instability 151, poor crosslinking efficiency, and the potential for product staining 152, 153. Despite the development of low-toxicity chemical crosslinkers, their residual byproducts may still present risks. As a result, physical crosslinking is gaining popularity as a simpler and safer alternative.

Radiation techniques, such as gamma 154, and electron beam irradiation 155, have been investigated as physical crosslinking methods. Notably, ultraviolet (UV) irradiation has been shown to induce covalent bond formation between collagen fibers 138, 156, 157. For instance, baking a scaffold at 105 °C for 24 hours in the presence of UV light has been demonstrated to achieve covalent crosslinking of collagen chains, thereby enhancing the scaffold's structural strength 135. Physical crosslinking is advantageous in producing highly biocompatible materials while avoiding the introduction of exogenous toxic chemicals into the tissue 158. As a result, physical crosslinking is increasingly recognized as a method for manufacturing biomaterials. However, these techniques may lead to protein denaturation and environmental contamination. Radiation-based crosslinking methods, however, face limitations including the need for specialized facilities, longer waiting times (due to the radiation half-life), and logistical challenges such as transportation 159. Furthermore, the crosslinking process may be uneven, leading to inconsistent results with each application 154.

Finally, biological crosslinking, particularly enzymatic crosslinking, has emerged as a promising alternative. Enzymatic crosslinking reactions are highly specific, rapid, and can be performed under physiological conditions, without introducing toxic or contaminating substances. Transglutaminase (TG), a multifunctional enzyme 160 found in animals, plants, and human tissues, has been widely used to crosslink ECM proteins 161. TG selectively mediates the formation of amide bonds between glutamine and lysine residues, facilitating cell adhesion through interactions with fibronectin and integrins 162. Another example is lysyl oxidase, which catalyzes the oxidative deamination of lysine and hydroxylysine residues in collagen and elastin, initiating the covalent crosslinking of ECM proteins 163. However, enzymatic crosslinking can sometimes lead to abnormal biochemical functions in collagen, with unpredictable consequences, such as those seen in diseases like diabetes 164. In addition, another study found that at low crosslinking concentrations, ECM treated with TG exhibited no significant strength increase 165.

## 8. Recent advances in DAT applications

DAT demonstrates promising adipogenic properties. However, it also faces several challenges, including limited angiogenic potential, rapid degradation, and insufficient mechanical properties. In contrast, composite hydrogels present distinct advantages over DAT hydrogels, such as the ability to incorporate pro-angiogenic cytokines or endothelial cells to enhance angiogenesis 123, 166, as well as the use of various crosslinking methods or the addition of other materials to improve mechanical strength 73, 144. However, the direct addition of bioactive factors presents several challenges, including high costs and susceptibility to inactivation. In contrast, the integration of these factors from the ECM helps to mitigate these issues. Additionally, John *et al.* demonstrated that DAT scaffolds seeded with ASCs induced early modulation of macrophage polarization compared to non-seeded DAT controls. However, longitudinal analysis revealed no significant differences between the groups in terms of macrophage phenotypic profiles, vascular perfusion, implant remodeling, or long-term host tissue integration 167. This study highlights the inherent challenges of cell-seeding therapies, particularly the rapid clearance of therapeutic cells, underscoring the need for strategies aimed at sustaining cell retention or protocols for replenishing cells to support therapeutic supplementation.

Recent studies on DAT-induced adipose tissue regeneration have shown heterogeneous adipogenesis following *in vivo* grafting, with some areas failing to undergo adipogenesis 64. Esteve *et al.* discovered that the adipose tissue ECM comprises septa and stroma. The stroma fibers, which are small and sparse, are enriched in the MSCA1+ adipogenic subpopulation, while the septal fibers are densely structured and contain MSCA1-/CD271- and MSCA1-/CD271high progenitors, which contribute to the differentiation and activation of myofibroblasts 65. Furthermore, a recent comparative analysis of abdominal adipose tissue layers revealed that superficial DAT had significantly higher mitochondrial density and enhanced adipogenic regenerative capacity compared to deep DAT, providing new insights into the adipogenesis effects of DAT and generating new directions for future research in adipose regeneration 49.

Adipose tissue has a highly developed vascular system that is essential for nutrient transport, waste metabolism, and the provision of paracrine signals for intercellular communication 21. Extracellular vesicles (EVs), as crucial paracrine mediators, play a key role in this process 168. Adipose tissue, functioning as an endocrine organ, also relies on EVs as carriers for intercellular signaling 169-171. However, previous studies have suggested that the decellularization process inevitably results in the loss of these vesicles. Consequently, future research should focus on improving cellular integration in implanted materials and restoring paracrine signaling functions.

In 2016, Luai *et al.* identified matrix-bound nanovesicles (MBVs) within the ECM and reported their presence in decellularized biological scaffolds 172. This groundbreaking discovery revealed that ECM scaffolds derived from tissues such as the urethral bladder, SIS, and dermis contained MBVs ranging in size from 10 to 1000 nm. These MBVs were resistant to various cell removal protocols, including chemical, enzymatic, and detergent treatments, and were shown to significantly alter cellular behavior. Remarkably, MBVs promoted macrophage polarization toward the anti-inflammatory M2 phenotype and influenced stem cell differentiation, phenomena previously linked to ECM-mediated structural remodeling. While MBVs share some similarities with exosomes (30-150nm) and microvesicles (150-1000nm), such as nanoscale size and miRNA content. While they lack typical molecular markers such as CD63, CD81, CD9, and Hsp70. Furthermore, Luai *et al.* demonstrated that MBVs from porcine urinary bladder matrix (UBM) and SIS were enriched in specific miRNAs 173. When these MBVs were applied to bone marrow-derived macrophages, the cells exhibited an anti-inflammatory phenotype, characterized by upregulation of M2 markers such as Fizz1 and Arg1, along with enhanced phagocytic activity and nitric oxide production. In contrast, inhibition of these miRNAs led to a shift toward a pro-inflammatory (M1-like) phenotype, marked by increased expression of iNOS and TNF-α. These findings suggest that MBVs mediate ECM-induced macrophage phenotype switching through their specific miRNA cargo, shedding new light on the immunomodulatory mechanisms by which ECM-based biomaterials support tissue repair. Further research by Van der Merwe *et al.* showed that MBVs from porcine bladder ECM could protect retinal ganglion cells from ischemic injury by reducing inflammation and providing neuroprotection, suggesting their potential in retinal and central nervous system diseases 174. Similarly, Anne *et al.* found that ECM hydrogels from porcine bladder ECM promoted the survival of hippocampal neurons and enhanced axonal growth. Additionally, MBVs from bladder ECM were taken up by neurons and promoted axon growth and branching, providing valuable insights for central nervous system injury repair 175. Although no studies have yet been conducted on MBVs derived from DAT, it is plausible that DAT-derived MBVs may also facilitate tissue regeneration and offer insights into ECM-mediated cellular behavior.

George *et al.* compared liquid-phase EVs with MBVs, finding that, although both had similar morphology and size, their miRNA profiles differed significantly 176. Specifically, 28 miRNAs were differentially expressed, with miRNAs associated with tissue development markedly enriched in MBVs. Additionally, MBVs exhibited a lipid composition rich in phospholipids associated with polyunsaturated fatty acids, lysophospholipids, and oxidized lipids, contrasting with the phosphatidylcholine-dominated composition of liquid-phase EVs. These differences suggest that MBVs play a significant role in ECM-mediated tissue repair and offer novel insights into ECM-based biomaterial design for minimally invasive therapies. Dake *et al.* employed click chemistry to conjugate the integrin α4β1 ligand LLP2A to the surface of electrospun scaffolds, facilitating the specific binding of EVs derived from placental mesenchymal stem cells 177. This functionalized scaffold mimicked natural ECM-EV complexes and was shown to enhance the migration of HUVECs, promote vascular sprouting, and upregulate angiogenesis-related genes such as KDR and TIE2. Additionally, LLP2A modification significantly improved EV adhesion to the scaffold surface, increased endothelial cell survival under hypoxic conditions, and inhibited apoptosis markers such as caspase 3 and caspase 9. These findings highlight the potential of integrin-mediated EV immobilization strategies in enhancing the vascularization capacity of tissue-engineered scaffolds, with promising applications in ischemic tissue repair. Dake's research provides important insights into adipose tissue regeneration, suggesting that future efforts in this field could benefit from the modification of DAT-derived MBVs to enhance adipose structural repair and functional regeneration.

## 9. Challenges and perspectives

Although the clinical application of DAT composite hydrogels is still in its early stages, their potential as acellular scaffold for adipose tissue regeneration or as a therapeutic delivery vehicle is significant 178. The manufacturing of DAT hydrogels as a biomaterial is relatively simple and does not raise significant ethical concerns. For instance, MTF Biologics currently markets Renuva™ in the U.S. as a decellularized human adipose-derived product for soft tissue augmentation. However, the exact mechanisms by which DAT hydrogel components influence cellular behavior are not fully understood. Current understanding the mechanisms proposes that ECM mediates cellular behavior through inherent surface morphology 179, biomechanical influences 180, and the impact of signaling peptides and proteins 181. However, the clinical translation of DAT hydrogels confronts numerous challenges 70. Primary among these is the complex decellularization process, which is difficult to standardize, leading to significant batch-to-batch variations that fail to meet ISO 13485 medical device quality control standards 182. Additionally, precise control over material properties, including mechanical performance, degradation rate, and drug release kinetics, remains chanllenges. Moreover, residual decellularization reagents may trigger immune reactions, while chemical crosslinking methods could generate toxic residues that impact biocompatibility 62. In large-scale production, crosslinking consistency and minimizing batch varations remain prominent issues, driving up production costs and complicating the manufacturing process. In terms of standardization and quality control, there is a lack of dedicated evaluation standards for DAT scaffolds. While multiple decellularization methods exist, low production efficiency impedes the progress of large-scale production. How to pre-screen adipose donors to collect clinically acceptable adipose tissue for raw materials, as well as addressing issues related to the use of xenogeneic adipose tissue-derived DAT, remain critical problems that need to be solved 70. Despite the therapeutic potential of DAT composite hydrogels, overcoming a range of technical, cost-related, and standardization challenges is essential to achieve a clinical-grade product comparable to Renuva™.

In recent years, significant progress has been made in the application of DAT composite hydrogels in adipose tissue regeneration, presenting new opportunities for clinical treatment. Currently, research on DAT composite hydrogels in tissue engineering mainly focuses on structural repair. However, future studies should place greater emphasis on promoting the functional regeneration of adipose tissue, particularly in terms of energy storage, vascularization, and the secretion of hormones and cytokines. In addition to direct incorporation of bioactive factors or cells to enhance adipose functional regeneration, 3D bioprinting technology has also provided important technical support for functional regeneration. 3D bioprinting technology includes two methods: Fused Deposition Modeling (FDM) and Stereolithography (SLA) 183. FDM works by heating thermoplastic filament to a semi-molten state, extruding it layer by layer, and allowing it to cool and solidify, making it suitable for printing high-strength scaffolds. For example, Temple *et al.* fabricated PCL scaffolds via FDM with 250 μm pores, populated them with HASCs, and demonstrated robust adipogenesis both *in vitro* and *in vivo* 184. FDM's high-temperature process prevents live cell printing, requiring scaffolds to be printed and subsequently seeded with cells. In contrast, SLA uses ultraviolet lasers to irradiate photosensitive resin layer by layer, providing higher precision and lower temperature, which allows the direct printing of cell-laden structures. For example, Pati *et al.* used SLA technology to print DAT hydrogel scaffolds containing HASCs, achieving excellent soft tissue regeneration and promoting adipogenic differentiation of stem cells 43. The advantage of FDM lies in its lower equipment cost and rich material options, although it has lower precision. SLA, on the other hand, has significant advantages in detailed structure and direct cell printing, though the equipment is expensive and requires post-processing 185. Both methods offer distinct advantages in adipose tissue engineering: FDM allows for printing scaffolds followed by cell seeding, while SLA can process cells and their microenvironment in a single step. In addition to these technologies, organoids based on DAT composite hydrogels hold promise for the treatment of metabolic diseases and adipose regeneration 168.

Future research should focus on the following key directions: (I) Optimizing decellularization methods, achieving efficient and non-toxic removal of cellular components while supporting large-scale production; (II) Extending DAT composite hydrogels degradation time, ensuring long-term cell survival and effective adipogenesis *in vivo*. This can be achieved by adjusting the crosslinking degree and material composition to design composites with controllable degradation rates that meet clinical demands; (III) Improving the mechanical properties of DAT composite scaffolds, ensuring they better simulate the biomechanical properties of soft tissue and prevent premature degradation or functional failure after implantation. This can be achieved by optimizing the molecular structure of the material and utilizing appropriate crosslinking technologies to enhance the mechanical properties and ensure its compatibility with the desired clinical applications; (IV) Investigating the impact of processing methods on DAT hydrogel properties, exploring how different decellularization methods influence the physical and chemical properties of hydrogels, and optimizing the preparation process to meet specific clinical applications; (V) Exploring the impact of DAT composite hydrogel components on cell behavior and tissue remodeling, utilizing organoid models to deeply investigate the cellular and molecular processes involved in adipogenesis and tissue repair, thereby providing a theoretical basis for improving therapeutic strategies; (VI) Ensuring the safety of DAT composite hydrogels, focusing on chemical composition, crosslinker residues, and immune responses to ensure their biocompatibility and clinical applicability, reducing potential risks in clinical applications; (VII) Strengthening the integration of functional regeneration and structural repair, optimizing the physical properties and biological functions of DAT composite hydrogels to enhance their multifunctional roles in soft tissue regeneration, such as angiogenesis and the secretion of bioactive molecules (MBVs), achieving comprehensive tissue regeneration effects; (VIII) Designing novel DAT composite hydrogels to address the evolving clinical needs, combining cutting-edge technologies and new challenges in clinical practice to develop more efficient, personalized therapeutic strategies that cater to the needs of different patient populations.

## 10. Conclusion

This review comprehensively presents fifteen years of advances in DAT composite hydrogels for adipose regeneration, critically evaluating historical developments, decellularization methodologies, biomaterial efficacy, and mechanical optimization strategies—particularly crosslinking-enhanced structural stability. While current research prioritizes structural repair, the critical gap in functional regeneration (metabolic activity, vascularization, endocrine signaling) fundamentally limits physiological relevance. Future innovation should concurrently advance structural fidelity and functional restoration to unlock the full therapeutic potential of DAT composite hydrogels platforms.

## Figures and Tables

**Table 1:** Decellularization methods (Methods A‒C and D‒G is porcine and human adipose decellularization workflow, respectively).

Step / Feature	Method A 28	Method B 28	Method C 28	Method D 11, 35	Method E 69	Method F 46	Method G 96
1	Thaw tissue	Thaw tissue	Thaw tissue	Freeze-thaw 3 times	Wash the adipose tissue	Thaw adipose tissue in NaCl buffer at room temperature	Store the lipoaspirate at -80 °C
2	0.02% Trypsin/0.05% ethylenediamine-tetraacetic acid (EDTA)	Digest collagenase (3 mg/g dry weight)	Digest collagenase (3 mg/g dry weight) + 0.05% EDTA	Incubate overnight in enzymatic digestion solution	1:1 mix adipose tissue/distilled water and centrifuge	Homogenization and dispersion	Thaw frozen tissues and wash with PBS for 2 h
3	3% Triton X-100 → 4% Deoxycholate	0.02% trypsin/0.05% ethylenediamine-tetraacetic acid 10 U/mL deoxyribonuclease	0.1% NP40 → 4% Deoxycholate → 1% SDS	Polar solvent extraction	Denature for 12 h in 4 M urea buffer containing protease inhibitor	Centrifugation, repeat the homogenization step and add 2M urea buffer	1% sodium dodecyl suladiposee (SDS) for 2 h
4	4% Ethanol + 0.1% Peracetic acid	10 U/mL lipase	0.9% NaCl + Tris-HCl + inhibitors	Incubate for 6 h in enzymatic digestion solution	Re-extract residues for 12 h with 4 M guanidine containing and centrifugation		Soak for 12-24 h in PBS solution containing 2.5 mM sodium deoxycholate
5	100% n-propanol and lyophilized	Lyophilization	Rinse three times and lyophilize	Wash 3 times (30 min each) in rinsing buffer solution	Filter and dialyze the supernatant	Centrifuge at 23, 000 ×g for 20 min at 4 °C and collect supernatant	Incubate in a solution containing DNase and RNase
Advan-tages	Complete decellularization, minimal lipid, high ECM retention	Simple process, efficient decellularization	Detergent-based, broad protein removal	Simple, strong lipid removal, injectable hydrogel	Good decellularization, hydrogel potential	Broad pathogen removal, low toxicity	Effective lipid removal, ECM retention
Disadvan-tages	More complex steps, multi-reagent, time-consuming	Lipid residue remains, lower GAG/growth factors	High DNA/lipid residue, poor ECM preservation	Harsh detergents, long duration	SDS may damage ECM	Lipid residue remains, ECM alteration	Long process, multi-enzyme, cost

**Table 2:** DAT-based composite hydrogels.

		Composite hydrogel system	Advantages	Disadvantages	Reference Citations
Cell-free scaffolds	DAT composite with natural materials	DAT/GAGs	Promote cell proliferation, adhesion, anticoagulation and wound repair High viscoelasticity, high water absorption capacity and high biocompatibility	Heterogeneous Batch-to-batch variability Biological contamination Limited changes in mechanical properties	23, 84
		DAT/MGC	Promote wound healing Increase the mechanical properties	Batch-to-batch variability Inadequately matched to soft tissue mechanical properties	67
		DAT/MAP	An ideal bio-adhesive Biocompatible Prevents the formation of abnormal collagen protofibrils Promotes wound healing Inhibits collagenase activity	Susceptible to oxidation Poor mechanical strength Poor long-term stability in air High material cost Potential biosafety issue	90
		DAT/SF	Good biocompatibility and degradability Adjustable structure and function	Poor adhesion and proliferation ability to neuronal cells Poor resistance to degradation Poor mechanical properties	96
		DAT/MC	An effective adhesive or thickener Inexpensive Good swelling ability and cell affinity High viscosity High biocompatibility	Poor mechanical properties	69, 95
	DAT composite with synthetic materials	DAT/PNIPAM	Promising thermally responsive smart polymers for biomedical applications	Non-degradable Lacks biologically active factors for cell adhesion and proliferation	90
		DAT/PEG	Mediates cell fusion Controllable mechanical properties	Inherently toxic Immunogenic Allergenicity	117, 186
		DAT/PCL	Controllable mechanical properties	Release of compounds toxic to cells during biodegradation Poor biocompatibility Fibrous encapsulation Inflammatory reactions	120
		DAT/PLCL			78
Complexed with cells or bioactive factors	DAT complexed with cells	DAT/HASCs	The potential for multidirectional differentiation Promote adipocyte regeneration Secrete a variety of cytokines	Encapsulated cells release excess hydrogen peroxide Leading to tissue damage	13, 124, 125, 187
		DAT/HUVECs	Secrete a variety of angiogenesis-promoting active factors with good vascular regeneration ability	Release excess hydrogen peroxide Limited adipose regeneration potential	123, 125, 187
		DAT/Human subcutaneous preadipocyte cells	Proliferate and differentiate mature adipocytes and increase the volume of adipose tissue	Limited adipose regeneration potential Limited differentiation into mature adipocytes	123
		DAT/ Dexamethasone	Dexamethasone and other glucocorticoids are necessary for the complete differentiation of adipose precursors as well as for the maintenance of key genes for glucose and lipid metabolism in cultured adipocytes and adipose tissue, and can increase adipogenesis	Short half-life	10
	DAT complexed with bioactive factors	DAT/EVs	Induces adipogenic differentiation of HASCs and promotes aortic endothelial cell proliferation, migration, and angiogenesis	The standardization, dose control, safety, and stability of EVs need to be considered	59, 127
		DAT/Angiogenic factor	Have natural pro-angiogenic effects	Short half-life, Potential tumorigenic risk, Complex production	41

**Table 3:** Young's modulus of composite hydrogels formed by DAT and natural materials.

Composite hydrogels	Young's modulus	Advantage
DAT+GAG	DAT+MGC/MCS 30-40 kPa 67	The overall mechanical properties of the composite hydrogel are adjustable, allowing it to simulate the physical characteristics of natural soft tissues and effectively prevent frictional irritation after implantation
	DAT+MCS 123 ±17 kPa 84	Enhancement of adipogenesis of MCS+DAT (8wt%DAT) composites
DAT+ Fibrin	22.4 ± 3.5 kPa 96	1:13 (v/v) DAT: Fibrin hydrogels with mechanical properties similar to natural adipose tissue enhanced vascularization both *in vitro* and *in vivo*
DAT+MC	3.8 kPa 69	The stiffness of DAT (6 wt%) and MC (6 wt%) hydrogels, similar to that of adipose tissue, promotes adipogenic differentiation of stem cells
